# Association between Older Age and Psychiatric Symptoms in a Population of Hospitalized Patients with COVID-19

**DOI:** 10.3390/jpm13060973

**Published:** 2023-06-09

**Authors:** Maria Camilla Cipriani, Cristina Pais, Vezio Savoia, Cinzia Falsiroli, Andrea Bellieni, Antonella Cingolani, Massimo Fantoni, Daniela Pia Rosaria Chieffo, Gabriele Sani, Francesco Landi, Giovanni Landi, Rosa Liperoti

**Affiliations:** 1 Fondazione Policlinico Universitario A. Gemelli IRCCS, Largo F. Vito 1, 00168 Rome, Italy; 2 School of Medicine, Università Cattolica del Sacro Cuore, Largo F. Vito 1, 00168 Rome, Italy

**Keywords:** older adults, COVID-19, psychiatric symptoms, delirium, hospitalization

## Abstract

Increased rates of anxiety and depression have been reported for older adults during the COVID-19 pandemic. However, little is known regarding the onset of mental health morbidity during the acute phase of the disease and the role of age as potential independent risk factor for psychiatric symptoms. The cross-sectional association between older age and psychiatric symptoms has been estimated in a sample of 130 patients hospitalized for COVID-19 during the first and second wave of the pandemic. Compared to younger patients, those who were 70 years of age or older resulted at a higher risk of psychiatric symptoms measured on the Brief Psychiatric Symptoms Rating Scale (BPRS) (adjusted (adj.) odds ratio (OR) 2.36, 95% confidence interval (CI) 1.05–5.30) and delirium (adj. OR 5.24, 95% CI 1.63–16.8)). No association was found between older age and depressive symptoms or anxiety. Age was associated with psychiatric symptoms independently of gender, marital status, history of psychiatric illness, severity of disease and cardiovascular morbidity. Older adults appear at high risk of developing psychiatric symptoms related to COVID-19 disease during hospital stay. Multidisciplinary preventive and therapeutic interventions should be implemented to reduce the risk of psychiatric morbidity and related adverse health care outcomes among older hospital inpatients with COVID-19.

## 1. Introduction

There is evidence that the COVID-19 pandemic has exerted a substantial impact on global mental health. Indeed, the high transmissibility and the uncertain prognosis that characterize the COVID-19 disease together with factors contributing to the social burden of the pandemic [1] such as enforced isolation, quarantine, widespread anxiety, and financial difficulties [2] may have influenced individual mental status.

A direct influence of the COVID-19 infection on the onset and progression of psychiatric disorders has also been explained on the basis of biology. A systemic inflammatory status characterizes the COVID-19 disease; elevated inflammatory biomarkers have been reported in several psychiatric conditions including depressive disorders, anxiety and sleep disturbances [3,4]. The brain may also be indirectly affected by COVID-19 through the host immune response to the infection [1,5,6,7].

Older age has been shown to be an independent risk factor for the COVID-19 disease severity and related mortality [8]. Yet, the role of age on increasing the risk of mental health disorders or psychiatric symptoms related to the COVID-19 pandemic has not been clarified.

There is evidence that compared to young adult and middle-aged patients, older adults showed higher resilience to the mental health effects of COVID-19, at least during the first wave of the pandemic [9]. In fact, although older adults are more likely to experience severe manifestations of COVID-19 and more likely to be socially isolated compared to younger individuals, lower rates of depression, anxiety, stress-related disorder, and suicidal ideation have been reported in this age group [10]. Several possible reasons are behind the observed higher vulnerability of young people to the mental impact of COVID-19. During pandemic, young adults and middle-aged individuals had to face several new challenges including job loss and financial problems, mourning, childcare, remote working and learning. Such conditions may have been responsible for the observed increased level of anxiety, depression and distress and decreased self-esteem. Older adults are less likely than younger individuals to experience unemployment and they may show higher ability to cope with stressful conditions. However, factors such as the lockdown experience, the fear of the disease, isolation and loneliness have had a negative impact on mental health of older adults leading to increased levels of anxiety and depression and accelerated progression of cognitive decline [11]. Although rates of anxiety and depression in older adults have resulted lower than in younger age groups during the pandemic, they have resulted higher compared to those of the pre-pandemic times [12]. The consequences of such burden of mental disorders are relevant. In fact, compared to individuals of the same age with no mental problems, older adults with psychiatric conditions are more likely to experience functional impairment and disability, and they are also at a higher risk of experiencing acute medical events, death and suicide [13].

To date, research has focused mainly on the investigation of the long-term effect of COVID-19 on mental health, and little is known regarding the onset of psychiatric morbidity during the acute stages of the disease. It is well known that older patients are likely to experience psychiatric symptoms and conditions during hospitalization, such as delirium, depression, and anxiety [14]. Such conditions increase the risk of negative health outcomes during hospitalization. For example, delirium is associated with increased risk of in-hospital morbidity (e.g., infections, falls), cognitive and functional impairment and disability, prolonged hospitalization, institutionalization, and death [15,16]. A deeper knowledge of the frequency and pattern of psychiatric morbidity during the acute stages of the COVID-19 disease and a better understanding of the role of age as potential risk factor for mental complications would help clarify some mechanisms behind the COVID-19-related excess mortality that has been observed in older adults as well as plan risk assessment programs and individualized therapeutic interventions during in-patient hospital care.

The aims of this study were to assess the prevalence and pattern of neuropsychiatric symptoms during the acute stages of the COVID-19 disease and to investigate the role of age as potential independent risk factor for such symptoms.

## 2. Materials and Methods

### 2.1. Study Design

We reported findings from a cross-sectional study including hospitalized patients for COVID-19 during the first and second waves of the pandemic, from April 2020 to January 2021.

The study was evaluated and approved by the Ethics Committee of the Catholic University of the Sacred Heart, Rome.

### 2.2. Sample

During the study time, 130 participants were enrolled among those patients consecutively admitted to the Columbus Covid Hospital in Rome, Italy.

Inclusion criteria were being at least 18 years of age and hospitalization with COVID-19 (the diagnosis of COVID-19 was determined in accordance with the World Health Organization interim guidelines).

Exclusion criteria were the presence of any diagnosed neurocognitive disorders, dementia or other neurodegenerative diseases that would have impaired the ability to participate or to provide informed consent.

### 2.3. Outcome Measures

Psychopathological assessment was performed within 5 to 10 days of hospitalization. Several neuropsychiatric conditions and symptoms were assessed in the study population using the following tools.

The NEECHAM confusion scale (NCS) was used to assess delirium. The NCS was developed for a rapid and bedside assessment of hospitalized patients. The scale evaluates components of cognitive status (attention, alertness, verbal and motor response, memory, and orientation), observed behavior and performance ability (general appearance and posture, sensory–motor performance, and verbal responses) and vital functions (vital signs). The scores range from 0 (minimal function) to 30 (normal function); the cut-off point is 24. The range from 0 to 24 points indicates the presence of delirium specifically; the 30–27 range indicates that the patient is non-delirious, 26–25 indicates that the patient is at risk, 24–20 indicates the presence of early to mild confusion, and 19–0 indicates moderate to severe confusion [17].

The Hamilton Depression Rating Scale (HAM-D) was used to assess the severity of depressive symptoms. The 21-item questionnaire was chosen for this study. The possible score range is 0–53; patients with scores of 0–7 are considered normal or in remission, scores of 8–16 suggest mild depression, 17–24 suggest moderate depression, and >24 indicate severe depression [18].

The Hamilton Anxiety Rating Scale (HAM-A) was used to measure the severity of anxiety symptoms. The scale consists of 14 items rating both psychic (mental agitation and psychological distress) and somatic anxiety (physical complaints related to anxiety). Each item is scored on a scale of 0 (not present) to 4 (severe), with a total score range of 0–56, with scores < 17 indicating mild severity, 18–24 mild to moderate severity, 25–30 moderate to severe anxiety, and >30 severe anxiety [19].

The Brief Psychiatric Rating Scale (BPRS) was used to assess psychopathology. It is a 24-item scale designed to rate several psychiatric symptom constructs. The symptom constructs include somatic concern, anxiety, depression, suicidality, guilt, hostility, elated mood, grandiosity, suspiciousness, hallucinations, unusual thought content, bizarre behavior, self-neglect, disorientation, conceptual disorganization, blunted affect, emotional withdrawal, motor retardation, tension, uncooperativeness, excitement, distractibility, motor hyperactivity, mannerisms and posturing. Items are rated on a 7-point Likert scale, from 1 “not present” to 7 “extremely severe”, with overall scores ranging from 24 to 168. The higher the scores, the more severe the psychopathology [20].

All the above tools were administered to participants by a dedicated psychologist using telephone or video call interviews.

### 2.4. Covariate Assessment

Patient demographic and clinical characteristics (age, sex, education, marital status, number of comorbidities, number of drug treatments prior to admission, and occupation) were extracted from the participants’ electronic medical records. Respiratory parameters were collected within 12 h of psychopathological evaluation. The respiratory parameters that were considered included partial pressure of oxygen in the arterial blood (PaO_2_), partial pressure of carbon dioxide in the arterial blood (PaCO_2_), peripheral oxygen saturations (SpO_2_), ratio of the partial pressure of oxygen in arterial blood (PaO_2_) to the inspired oxygen fraction (FiO_2_). PaO_2_ and PaCO_2_ were obtained from an arterial blood gas analysis performed by a physician [21].

The participants’ frailty status was assessed using the Clinical Frailty Scale (CFS) [22]. The CFS is a simple and intuitive judgement-based frailty tool that evaluates specific domains including comorbidities, function, and cognition to produce a frailty score ranging from 1, “very fit”, to 9, “terminally ill.” The Activities of Daily Living (ADL) scale [23] and the Instrumental Activities of Daily Living (IADL) scale [24] were used to assess disability status. The ADL scale is based on 6 levels of self-performance in basic activities of daily living: dressing, eating, toilet use, bathing, mobility, and urinary and fecal continence. The IADL scale is based on 8 levels of self-performance in instrumental activities of daily living: meal preparation, housework, cleaning, managing finances, phone use, shopping, transportation, managing medications. Higher scores correspond to higher levels of autonomy.

### 2.5. Statistical Analysis

The entire study population was divided into two groups according to age. Specifically, older adults were defined as those aged 70 years or older and adults included those aged younger than 70 years. The cut-off value of 70 years of age was chosen to be in line with the cut-off level of the age conventionally established by the Italian Ministry of Health to be used in geriatric research by scientific institution for research, hospitalization and health care (IRCCS) in Italy.

Descriptive statistics for all variables examined were calculated in the two study groups. Specifically, categorical variables were synthetized and presented using proportions; means and corresponding standard deviations were used to present continuous variables. Chi-square tests and unequal variance *t*-test were used to compare categorical and continuous variables, respectively.

Logistic regression models were used to estimate the potential association between age and presence of psychiatric symptoms. We used four different logistic regression models to explore the association between age and (a) psychiatric symptoms at the BPRS, (b) depressive symptoms at the Hamilton Depression Rating Scale, (c) anxiety at the Hamilton Anxiety Rating Scale, and (d) delirium at the Neecham confusion scale. We defined the presence of significant psychiatric symptoms as a score at the BPRS equal to or higher than 31, which is the median value of the BPRS score distribution in the study sample (range of 24–49). Variables for which the differences in the distribution across the levels of age resulted statistically significant and those variables which were considered clinically related to both age and psychiatric symptoms were included in the logistic models as covariates. From these models, odds ratios (ORs) and the corresponding 95% confidence intervals (CIs) were estimated after adjusting for potential confounders. All analyses were performed using SAS software V9.

## 3. Results

Overall, 130 hospitalized patients with COVID-19 were included in the present study. Socio-demographic, functional, and clinical characteristics of the participants are reported in Table 1. With respect to age, the sample included 47 (36%) older adults (individuals aged 70 years or older, age range 70–92), and 83 (64%) adults (individuals younger than 70 years, age range 26–69). The mean age for the elderly individuals was 77.7 ± 6.2 years, and that for the adult individuals was 52.4 ± 10.9.

In both age groups, about two thirds of the participants were female and the majority were married. Most participants were community dwelling, while only 12% of the older individuals lived in nursing homes.

As expected, more than half of the older adults were found to be frail according to the CFS. Bilateral pneumonia was found to be prevalent in younger individuals (75.3% in adults versus 67.4% in older adults), but respiratory failure was prevalent in the elderly subsample (for over 50% of older adults, a p/f (PaO_2_ divided by FiO_2_) of <300 was reported).

Among the neuropsychiatric conditions explored, delirium was prevalent in the group of older adults (31.9% versus 8.3% in adult individuals). History of psychiatric illness was more frequent in adult (15.7%) than in older individuals (10.6%). There were no significant differences in the prevalence of anxiety or depressive symptoms between the two groups. The mean BPRS score was higher in the group of older adults compared to that in the younger patients (34.0 versus 31.1).

As expected, cardiovascular comorbidities including hypertension, ischemic heart disease, diabetes, dyslipidemia, and heart failure resulted more frequent in the group of older adults compared to that of the adult individuals.

The prevalence of psychotropic medication use was less than 10% in the overall sample, with older adults displaying the highest use.

Finally, for older adults, longer duration of hospitalization was documented compared to the adult participants (33.0 versus 21.7 days, respectively).

Table 2 summarizes the distribution of psychiatric symptoms measured at the BPRS. The most frequently observed symptoms in both groups included anxiety, depression, and blunted affect. Among the 24 symptoms examined, only exaggerated self-esteem and motor tension were found to be differently distributed in the two groups, with the former prevalent in older individuals and the latter in adult subjects. Slight differences were also observed between the two groups. Such differences included an increased prevalence of anxiety, depressive symptoms and blunted affect in the adult group compared to the older adults and higher prevalence of disorientation, self-neglect and uncooperativeness in the older group compared to the adult individuals.

Table 3 reports the results from logistic regression models estimating the association between age and psychiatric symptoms at the BPRS, depressive symptoms, anxiety and delirium. After adjusting for potential confounders including gender, marital status, respiratory failure (p/f < 300), bilateral pneumonia, history of psychiatric illness, cardiovascular comorbidities (hypertension, ischemic heart disease, heart failure, diabetes, and dyslipidemia), the age of over 70 years was significantly associated with the occurrence of psychiatric symptoms defined as a score of 31 (median value of the distribution) or higher at the BPRS (OR 2.36; IC 95% 1.05–5.30) and delirium (OR 5.24; CI 95% 1.63–16.8). No association was found between advanced age and depressive symptoms or anxiety.

## 4. Discussion

The present study showed that among the hospitalized patients with COVID-19, those who were 70 years of age or older resulted at higher risk of psychiatric symptoms and delirium. Age appeared to be a risk factor for the development of psychiatric symptoms regardless of gender, marital status, history of psychiatric illness, severity of illness, and cardiovascular morbidity. Among the psychiatric symptoms and conditions investigated, anxiety and depression were the most frequently reported. However, no significant difference in the prevalence of such conditions was observed with respect to age.

Several studies have reported higher rates of psychiatric morbidity, including depression and anxiety, during the COVID-19 outbreak and lockdown time among young and adult individuals, especially women, compared to older adults [25]. For example, a meta-analysis of studies from the first year of the pandemic demonstrated that despite higher rates of somatic comorbidity and mortality among older adults compared to young individuals who have experienced COVID-19, psychiatric conditions such as anxiety, depression, and distress were significantly increased among young patients [26]. Similarly, a large-scale survey promoted by the US Center for Disease Control (CDC) found an increased prevalence of anxiety and depression among young adult patients [27]. High levels of resilience during difficult times characterize older adults. Such protective mechanism, despite the experienced social isolation, the severity of symptoms and the reduced access to health care, may have been the main reason for the lower psychological distress related to COVID-19 that has been systematically observed in the geriatric population [28,29].

Our study has specifically investigated the relation between COVID-19 and psychiatric morbidity during the acute stages of the disease among hospital inpatients. COVID-19 seems to exert a differential impact on mental status across age groups, and also in the most acute stages of the disease in older adults appearing the most likely ones to develop psychiatric symptoms. The specific moment of observation and the hospital setting may explain the reason of such inverted role of age on the risk of mental health disorders compared to what was observed during the pandemic so far.

Several mechanisms may explain the age-related vulnerability to develop psychiatric symptoms among hospitalized patients with COVID-19. The acute respiratory syndrome with fever and respiratory distress represents the typical clinical presentation of COVID-19. However, it is well known that COVID-19 is a multi-organ disease, and about one-third of patients develop neuro-psychiatric symptoms during the acute phase [30]. Biological factors such as the potential neurotropism of the virus and the pro-inflammatory cytokine cascade are likely to be involved [31]. Somatic comorbidities, nosocomial infections and medications are well-known risk factors for psychiatric symptoms and delirium in older adults who are admitted to a hospital for any reason. Physical and cognitive frailty that may have characterized the pre-morbid status of older patients may also be recognized as factors potentially influencing the risk of psychiatric symptoms during hospitalization [14,32]. Finally, chemical and physical restraints eventually used to limit physical consequences of agitation, aggression, and other symptoms may have increased the severity of such manifestations and the probability of developing additional psychiatric disturbances [14].

As expected, among the neuropsychiatric conditions that have been investigated in this study, the prevalence of delirium was significantly higher in the older age group compared to that of the younger individuals. This is not surprising because delirium is one of the most frequent syndromes among older hospital inpatients, especially those having surgery or being admitted to intensive care units. Indeed, age is recognized as one of the strongest risk factors for delirium. In several studies, delirium was also identified as a possible presenting manifestation of COVID-19 in older adults, even in the absence of other relevant symptoms [33]. The same pathophysiologic mechanisms underlying delirium due to other etiologies can be postulated for COVID-19-related delirium. The systemic inflammatory and hypoxic–ischemic status induced by the respiratory disease may work as a trigger factor acting upon a background of multiple potential risk factors for delirium including age, frailty, cognitive impairment, multimorbidity and polypharmacy that make older patients extremely vulnerable to develop the syndrome. Similarly, to what has been largely documented in other conditions, also among COVID-19 patients, delirium has been associated with longer duration of intensive care unit and hospital stay, higher probability of being discharged with disability, and higher mortality rates [34]. The onset of delirium among older patients may have to some extent influenced the excess mortality related to COVID-19 in the geriatric population.

Integrated care approach has been indicated as the potential most effective strategy to deliver care to older adults with COVID-19 [35]. Such model is based on care co-managed by a multidisciplinary team that may target the multiple needs of older adults, especially those who are the most complex patients and present with advanced age, physical and cognitive frailty, multimorbidity and polypharmacy. The identification of risk factors for developing psychiatric symptoms and delirium during the hospital stay and the early diagnosis of new psychiatric disturbances are extremely relevant to design and implement prevention programs and therapeutic interventions that are tailored to the individual patients. For example, promoting early mobilization of patients by nurses and physical therapists in respect to the needed measures of isolation, implementing the use of smart phones and tablets to ensure daily video contacts between patients and their families, and conducting regular revisions of prescribed medications dedicating special attention to those ingredients that may have psychotropic adverse effects represent some of the potential interventions that can be put in place to favor orientation, cooperation and to reduce the risk of these patients developing psychiatric symptoms and delirium. Based on the evidence from this study, those who are tasked with planning and delivering care in potential scenarios such as the COVID-19 pandemic should take into account the fact that older age carries an additional risk of morbidity and mortality that may be at least in part related to acute psychiatric complications. The adequate management of such complications may be beneficial for patients in terms of improved morbidity, quality of life and survival.

The present study has several limitations. The cross-sectional design of the study does not allow to establish temporal relationships and causal links for the observed associations. The assessment of psychiatric symptoms has been based on telephone and video-call interviews and therefore some of the collected information may have been misclassified [36,37]. Although the validity of the BPRS as a transdiagnostic assessment tool has been recently reported, this scale was originally developed to detect psychopathological symptoms in psychotic disorders and therefore it may not be adequate to identify symptoms in individuals without known psychiatric conditions [20]. Such consideration may also explain the failure of observing a direct correlation between scores at HAM-A and HAM-D scales and the identification of anxiety and depressive symptoms at the BPRS. Finally, the findings refer to a sample of patients hospitalized for the COVID-19 disease during the first and second waves of the pandemic, and they cannot be generalized to other populations.

In conclusion, our study showed that age is a strong risk factor for developing psychiatric symptoms during hospitalization for COVID-19. As it occurs in numerous acute diseases leading to hospitalization, age is also a strong risk factor for delirium, a complex geriatric syndrome that may determine increased disability and mortality even after recovering from an acute disease. Preventive and therapeutic interventions based on a multidisciplinary approach that takes into consideration the multiple and specific needs of the individual should be designed and implemented for older in-hospital COVID-19 patients.

## Figures and Tables

**Table 1:** Socio-demographic, functional, and clinical characteristics of study population according to age.

	OLDER ADULTS 70 Years of Age or Older (n = 47) (%)	ADULTS Less than 70 Years of Age (n = 83) (%)	*p*-Value
Age (mean ± sd)	77.7 ± 6.2	52.4 ± 10.9	<0.0001
Female	61.7	63.8	0.8069
Marital status - Married - Single - Widowed - Divorced	66.7 2.2 24.4 6.7	54.2 25.3 1.2 16.9	0.1721 0.0010 <0.0001 0.1044
Living Condition - Community dwelling - Nursing home resident - Other living facility	80.5 12.2 7.3	94.8 2.6 2.6	0.0361
Pre-admission Frailty status - Fit (CFS = 1–3) - Very mild frailty (CFS = 4) - Living with mild to very severe frailty (CFS 5–8)	46.8 8.5 44.7		
Bilateral pneumonia	67.4	75.3	0.3372
Respiratory failure p/f < 300	51.1	35.7	0.0869
Delirium (NCS < 24)	31.9	8.3	0.0005
Anxiety symptoms (HAM-A > 17)	23.4	21.4	0.7939
Depressive symptoms (HAM-D > 10)	44.7	51.2	0.4747
BPRS overall score	34.0	31.1	0.0073
History of psychiatric diseases	10.6	15.7	0.4255
Hypertension	74.6	39.0	0.0001
Ischemic heart disease	29.8	6.1	0.0003
Heart failure	21.7	0.0	<0.0001
Diabetes	27.7	17.1	0.1549
Dyslipidemia	14.9	6.1	0.0979
Psychotropic drug use: - Benzodiazepines - Antidepressants - Antipsychotics	8.7 6.5 4.3	1.2 3.6 1.2	0.0347 0.4526 0.2566
Length of hospital stay (days) (mean ± sd)	33.0	21.7	0.0043

**Table 2:** Pattern of psychiatric symptoms at the BPRS according to age.

	OLDER ADULTS 70 Years of Age or Older (n = 47) (%) *	ADULTS Less than 70 Years of Age (n = 83) (%) *	*p*-Value
Somatic Concern	36.2	47.6	0.2049
Anxiety	70.2	80.9	0.1606
Depression	59.6	64.3	0.5930
Suicidality	2.13	2.38	0.9259
Guilt	34.0	39.3	0.5519
Hostility	23.4	27.4	0.6185
Elated mood	4.3	5.9	0.6787
Grandiosity	14.9	4.8	0.0449
Suspiciousness	14.8	15.5	0.9291
Hallucinations	4.3	5.9	0.6787
Unusual thought content	2.1	1.2	0.6748
Bizarre behavior	4.3	1.2	0.2607
Self-neglect	10.7	3.6	0.1052
Disorientation	4.3	1.2	0.2607
Conceptual Disorganization	2.1	0.0	0.1796
Blunted affect	46.8	55.9	0.3147
Emotional withdrawal	23.4	25.0	0.834
Motor retardation	12.8	11.9	0.8852
Tension	12.8	27.7	0.0492
Uncooperativeness	11.9	4.3	0.1455
Excitement	23.4	29.7	0.4343
Distractibility	2.1	2.4	0.9259
Motor hyperactivity	2.1	7.1	0.2209
Mannerisms and posturing	0.0	0.0	-

* Percentage of subjects with a score of 2 or higher for individual symptoms at the BPRS.

**Table 3:** Association between age and psychiatric symptoms (BPRS > 31, 50th percentile of the distribution), depressive symptoms (HAM-D > 10), anxiety (HAM-A > 17), delirium (NCS < 24) (reference group: individuals aged less than 70 years).

	Crude OR	Adj. * OR	95% CI
Psychiatric symptoms (BPRS > 31)	2.00	2.36	1.05–5.30
Depressive symptoms (HAM-D > 10)	0.92	0.96	0.43–2.13
Anxiety (HAM-A > 17)	1.10	1.12	0.44–2.84
Delirium (NEECHAM < 24)	6.01	5.24	1.63–16.8

* adjusted for gender, marital status, respiratory failure (p/f < 300), bilateral pneumonia, history of psychiatric diseases, cardiovascular comorbidities including hypertension, IHD, HF, diabetes, dyslipidemia.

## Data Availability

The data presented in this study are available on request from the corresponding author. The data are not publicly available due to privacy restrictions.
